# Troubleshooting Transvenous Pacemakers with Point of Care Ultrasound (POCUS)

**DOI:** 10.24908/pocusj.v10i01.18073

**Published:** 2025-04-15

**Authors:** Emily Gohde, Seth Lotterman, Ikram Irfanullah, David Hansen, Felix Pacheco, Adam Wise, Matthew Tichauer, Trent She

**Affiliations:** 1 Department of Emergency Medicine, University of Connecticut, Farmington, CT, USA; 2 Department of Emergency Medicine, Hartford Hospital, Hartford, CT, USA

**Keywords:** point of care ultrasound, POCUS, arrhythmia, critical care

## Abstract

Emergent transvenous pacing can be performed for patients with an unstable bradyarrhythmia in the emergency department. While emergent transvenous pacemaker (TVP) placement is performed relatively rarely, its importance in the management of these patients requires emergency physicians to be familiar with the procedure. Point of care ultrasound (POCUS) is traditionally used for the initial vascular access associated with TVP placement but can also be indispensable in the subsequent placement and advancement of the TVP wire itself. This article discusses two cases and then outlines a general protocol that incorporates POCUS into a standard emergent TVP procedure. Further, we outline some common POCUS troubleshooting tactics to improve success and ways in which POCUS can rapidly identify complications.

## Introduction

Point of care ultrasound (POCUS) has become the standard of practice to facilitate many procedures in the emergency department. Its use in the placement of emergent transvenous pacemakers (TVPs) has been explored mostly in case reports and case series [1,2]. However, there are a few studies that have looked at POCUS use in a cardiac catheterization lab or an emergency department setting [3,4]. TVP placement itself is a relatively rare but important stabilizing procedure in patients with symptomatic bradycardia refractory to medical treatment. Traditionally, proper pacemaker placement is confirmed indirectly via telemetry by visualizing the presence of a new left bundle branch block (LBBB), characteristic pacemaker spikes, as well as a sharp increase in a patient's heart rate. However, this technique can be imprecise. The presence of these telemetry findings can occur despite the TVP wire being in an improper location and absence of these telemetry findings can represent not only a mispositioned wire but also improper conduction or insufficient voltage. If the wire is in an incorrect location, telemetry does not indicate the location of the wire and gives little suggestion on how to then guide the TVP wire into the apex of the right ventricle. The inability to visualize the TVP wire can result in the traditional placement being more prone to complications and ultimately, may result in failure of the procedure, harm to the patient, or delay in bradycardia management [5,6].

Direct visualization of the pacemaker wire passing directly into the apex of the right ventricle to confirm proper location of the TVP wire is a significant benefit of POCUS. The TVP wire appears as a highly echogenic structure that can be easily seen in the right atrium and ventricle when properly placed. Should a TVP wire not be seen in the right side of the heart despite proper depth, POCUS can be used to identify other possible locations of the TVP wire. Based on this knowledge, clinicians may redirect the wire to its appropriate location. Below, we discuss two cases that illustrate the utility of POCUS in troubleshooting unsuccessful or difficult TVP placements. Thereafter, we describe a workflow for future TVP placements and offer some troubleshooting tips.

## Case 1

An 85-year-old man with a history of atrial fibrillation, congestive heart failure, and pulmonary fibrosis presented to the emergency department after being found unresponsive by paramedics with a heart rate of 20-30 bpm. The patient had initially called emergency medical services with complaints of dizziness and nausea. Prehospital providers administered atropine, glucagon, and ondansetron, and started an epinephrine infusion for symptomatic bradycardia and hypotension. Prehospital transcutaneous pacing was attempted but there was poor capture, even with maximum electrical output, and it was subsequently discontinued. On arrival to the emergency department, his vitals were significant for a blood pressure of 167/71 mmHg, heart rate of 38 bpm, temperature of 95.6F, respiratory rate of 18 and an oxygen saturation of 97% on 2 liters of oxygen via nasal cannula. A review of the patient's medication list showed that he was not taking any antiarrhythmics or nodal blocking agents. An electrocardiogram showed the patient was in complete heart block. Although the patient's mental status improved significantly after prehospital interventions and his blood pressure was stabilized, a decision was made to pursue emergent TVP placement. This was given his poor response to medical treatment and requiring vasopressor administration to maintain an adequate heart rate. Cardiology was consulted as emergency department providers prepared for TVP placement.

**Figure 1.**
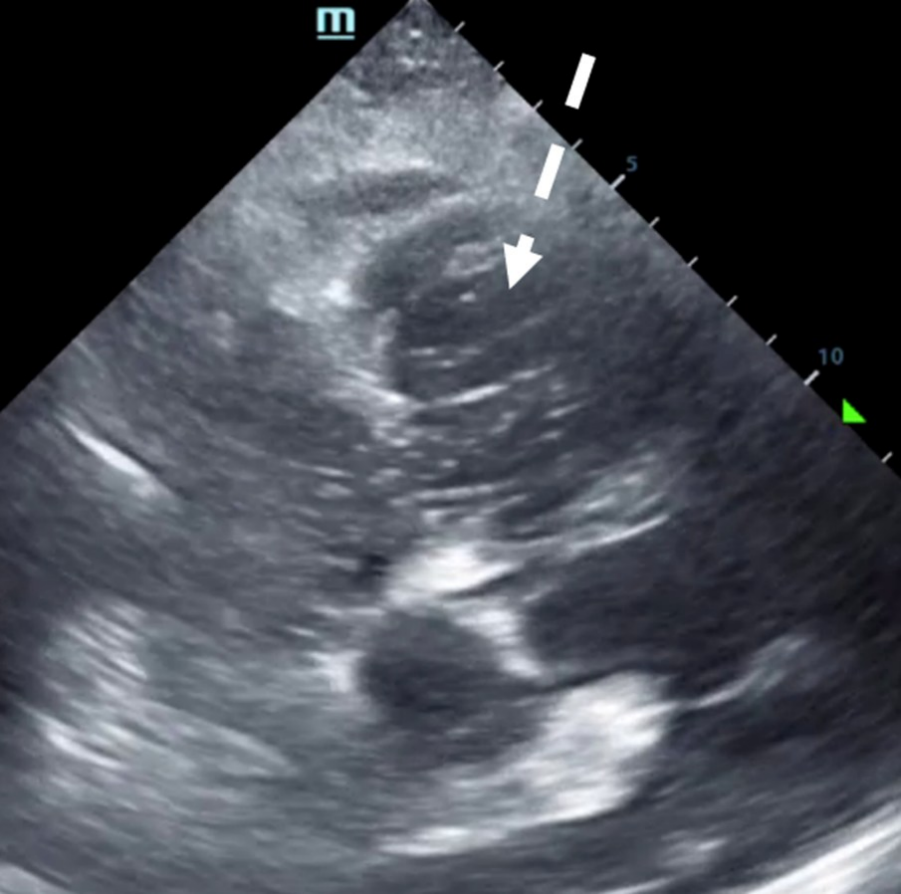
Subxiphoid cardiac point of care ultrasound (POCUS) view. Post-infusion of agitated saline with echogenic bubbles noted within the right ventricle (dotted white line) confirming venous placement of central venous catheter (CVC). Also see Supplemental Video 1.

A 7-French single lumen central venous catheter (CVC) was successfully placed in the right internal jugular vein without complication. A TVP wire was then introduced via the CVC and advanced slowly to a depth of 40 cm. The pacing generator was turned on with an output of 5 mA. Unfortunately, despite incremental increases in the electrical current, no LBBB or pacemaker spikes were noted on telemetry monitoring. Moments later, the patient's right arm began to spontaneously twitch in concordance with the rate of transvenous pacing. The pacing generator was turned off and the pacer wire was withdrawn, which stopped the patient's right arm twitching. A second advancement of the TVP wire was attempted and caused a similar event. At the time, clinical suspicion was that the TVP wire was being advanced towards the right arm through a subclavian vessel. The wire was withdrawn again and a second emergency department provider utilized POCUS to confirm venous placement of the CVC via an infusion of 10 cc of agitated saline (Figure 1). After the saline was infused, echogenic material was noted in the right side of the heart on a subxiphoid cardiac POCUS, confirming the CVC was in the venous system. A third attempt was then made to correctly place the TVP wire, this time under POCUS guidance.

**Figure 2.**
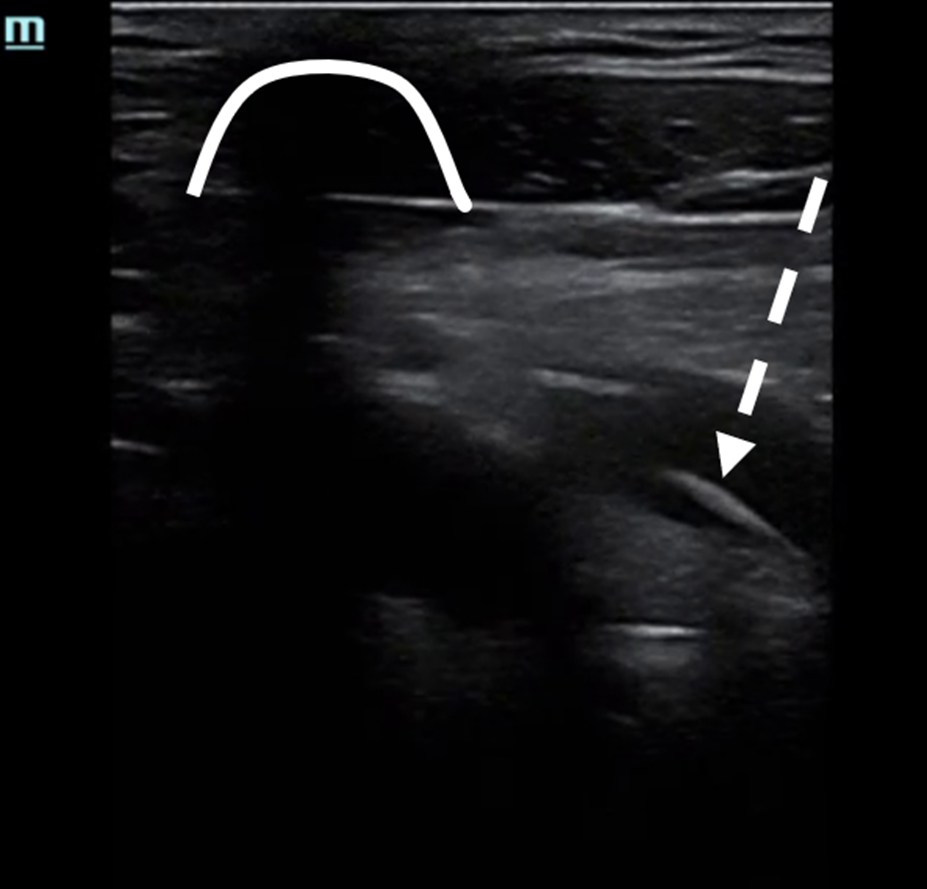
Infraclavicular point of care ultrasound (POCUS) view of the subclavian vein with linear echogenic material within the vein (dotted white line), representing a pacemaker guidewire. The clavicle is seen superiorly in this view (solid white line). See Supplemental Video 2.

**Figure 3.**
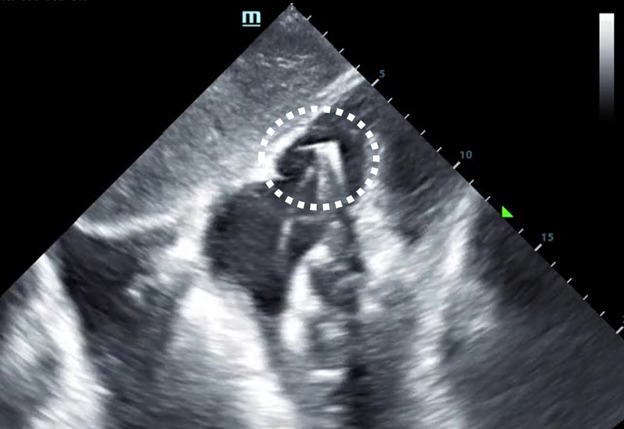
The tip of the transvenous pacing wire is seen advancing into the right ventricle past the tricuspid valve (white dashed circle) in this subxiphoid cardiac point of care ultrasound (POCUS) view.

As the TVP wire was advanced to a depth of 40 cm, a simultaneous subxiphoid cardiac view was obtained which did not visualize the wire in the right-side of the heart. An infraclavicular view of the distal subclavian vein with a linear probe was then obtained, demonstrating the echogenic wire in the right subclavian vein (Figure 2). The wire was withdrawn and given additional leftward curvature by the first emergency department provider. On this fourth and final attempt, the wire was seen under POCUS advancing into the apex of the right ventricle (Figure 3). Electrical capture was confirmed at a heart rate of 80 bpm. Shortly after the TVP was stabilized, the epinephrine infusion was discontinued. The patient remained normotensive with normal mentation throughout the procedure. He was ultimately admitted to the cardiac care unit and underwent a permanent dual-chamber pacemaker placement shortly after hospital admission. He was later discharged in good health.

## Case 2

A 79-year-old woman with a history of atrial fibrillation was found by her family unresponsive at home. Her husband called emergency medical services, and she was transferred to an outside hospital where she was found to be unresponsive, hypotensive, and bradycardic with a heart rate of 20 bpm. She was intubated, transcutaneous pacing was initiated, and she was started on a norepinephrine infusion. She later arrived at our hospital actively receiving transcutaneous pacing. Her vitals upon arrival included a blood pressure of 109/58 mmHg, a heart rate of 80 bpm, a temperature of 87F, a respiratory rate of 14 and an oxygen level of 96% on the ventilator. Her medication list included metoprolol, prescribed for her atrial fibrillation. As the patient remained on transcutaneous pacing and a norepinephrine infusion, the decision was made by the emergency department team to place an emergent TVP.

**Figure 4.**
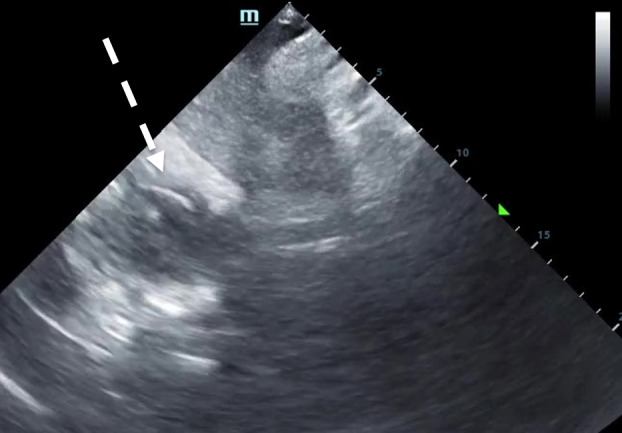
A sagittal subxiphoid view showing the transvenous pacing wire curled in the right atrium (white dashed arrow). See Supplemental Video 3.

A 7-French CVC was placed in her right internal jugular vein without difficulty. The TVP wire was slowly advanced to a depth of 40 cm. Unfortunately, no electrical capture was noted, despite the electrical current being increased to 10 mA from the TVP generator. A second provider immediately obtained a subxiphoid view of the heart with POCUS showing an echogenic pacing wire that had curled around in the right atrium without traversing through the tricuspid valve (Figure 4). The TVP wire was removed and had its curvature straightened, after which the wire was re-advanced into the CVC for a second attempt. On a simultaneous subxiphoid view, the TVP wire was seen in the right atrium and noted to pass through the tricuspid valve into the apex of the right ventricle. The patient's electrical capture was noted, their heart rate was set to 70 bpm, and they were ultimately admitted to the intensive care unit. No obvious cause was found for the patient's bradycardia and after several days with the patient's intrinsic heart rate in the 70-80s, the TVP was discontinued and she was discharged to a rehab facility.

## The Addition of POCUS into Standard Transvenous Pacing Procedure

**Figure 5.**
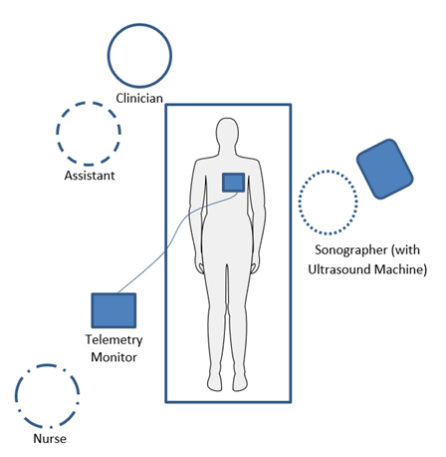
Recommended positioning of the patient room for temporary transvenous pacing. The clinician (blue solid circle) and the assistant (blue dashed circle) should be at the head of bed on the patient's right side to perform the procedure while the sonographer (blue dotted line) should be on the patient's left side to obtain optimal cardiac clips. A nurse should be in the room (blue dotted and dashed line) to operate the portable telemetry monitor with pads attached to apply transcutaneous pacing if needed.

In both above cases, POCUS provided invaluable information about the positioning of the TVP wire and greatly aided in the adjustments needed to successfully complete the procedure. Although POCUS is traditionally utilized solely for the placement of the CVC prior to advancement of the TVP wire, our suggestion is that POCUS be utilized in the placement of the TVP wire as well. In addition to assessing the patient post-procedurally for proper TVP placement, POCUS can be used for real-time assessment of the TVP wire location. We suggest having three emergency department staff members dedicated to this procedure, rather than the traditional two. These include 1) a clinician, who would physically perform both the CVC insertion and placement of the wire under sterile technique, 2) an assistant, not operating under full sterile technique, who would operate the pacemaker generator (and perhaps even the TVP balloon) at the direction of the proceduralist, and 3) a sonographer, also not operating under full sterile technique, who would operate the ultrasound machine and obtain cardiac POCUS and vascular views after the proceduralist completes CVC placement (Figure 5). We refer to these three staff members as the clinician, the assistant and the sonographer respectively during the rest of this section. With proper planning and review prior to the procedure, only one clinician is needed. Use of the pacemaker generator (and balloon) can be reviewed with the assistant, and the best window for cardiac POCUS and the location of subsequent vascular POCUS can be located by the clinician and communicated to the sonographer prior to initiating the procedure. However, having an experienced sonographer would certainly improve the timeliness and quality of the cardiac views.

The traditional pre-procedural setup for TVP placement should be observed with a few minor modifications. First, the preparation, cleaning and draping of the traditional right internal jugular vein access site may be extended to include the distal clavicle for sonographic access to the right subclavian vein. This may require modifying the plastic opening of the drape. Second, the sterile drape should be placed to allow access to the patient's subxiphoid space. This can be accomplished by lifting and securing the drape to a medical pole. Third, the screens of both the telemetry monitor and the ultrasound machine should ideally be visible to the three staff members involved with the procedure, or at the very least, to the clinician and the sonographer.

After completing pre-procedural setup, the clinician should place the initial CVC under ultrasound guidance with sterile technique. Once this CVC is placed utilizing the linear probe, control of the ultrasound can be relinquished to the TVP placement sonographer who will switch to the phased array probe. The linear probe should be left with sterile probe cover on the sterile procedural field. Once the sonographer transitions to the phased array probe, a subxiphoid or apical 4-chamber view should be obtained. There are other, less frequently used windows to visualize the right-side of the heart. However, these cardiac POCUS windows (i.e., subxiphoid or apical 4-chamber) are familiar to most emergency department physicians and have adequate views of the right atrium and right ventricle that will allow for the most direct view of the TVP wire passing into the heart. The clinician can then infuse a flush of agitated saline and observe for echogenic material in the right-side of the heart. By doing so, the CVC is both confirmed to have a patent lumen and to be in the appropriate venous circulation. If no agitated saline is visualized in the right-side of the heart, control of the ultrasound can be returned to the clinician and the CVC can be adjusted or replaced using the sterile linear probe.

Once placement of the CVC is confirmed, the clinician can focus on appropriately placing the pacemaker wire into the right ventricle. After ensuring the sheath is attached to the distal end of the CVC, the pacemaker wire can be slowly advanced to a depth of 20 cm. Next, the balloon should be inflated (either under sterile technique by the clinician or by ceding control of the 3 cc syringe to the assistant for inflation of the balloon). The pacing generator may be turned on at this time. For our initial settings, we recommend a heart rate of 60-80 bpm, an output of 10 mA and a sensitivity of 2 mV, but these can be adjusted to the preferences of the clinician. As the clinician slowly advances the wire, the sonographer should maintain an adequate sonographic view of the heart, paying particular attention to the right atrium and ventricle. At 20 cm, the wire may be in the lumen of the right atrium and be clear as an echogenic structure on POCUS, but more advancement of the wire may be needed [7]. We recommend slowly advancing the wire up to a depth of 40 cm. As the clinician advances the pacemaker wire, the sonographer should visualize the wire in the right-sided chambers of the heart and share the views with the clinician, allowing for real-time information on the location of the pacing wire. The wire can then be properly followed via POCUS as it floats into the correct location at the apex of the right ventricle. The assistant can then turn on the pacing generator.

## Troubleshooting Recommendations with POCUS

*If the pacemaker wire is not visualized at 40 cm on cardiac POCUS*

If the pacing wire is not seen on an initial cardiac POCUS with a depth of 40 cm, careful advancement to a depth of 50 cm may be considered. However, between this 40-50 cm depth, POCUS can also be used to evaluate for several venous locations where the pacing wire may have inadvertently been advanced. These locations include the inferior vena cava (IVC), the subclavian vessels, and the contralateral internal jugular vein. If the pacing generator is on, pacing may occur if the wire is in the IVC but is unlikely if the wire is in the neck vessels.

**Figure 6.**
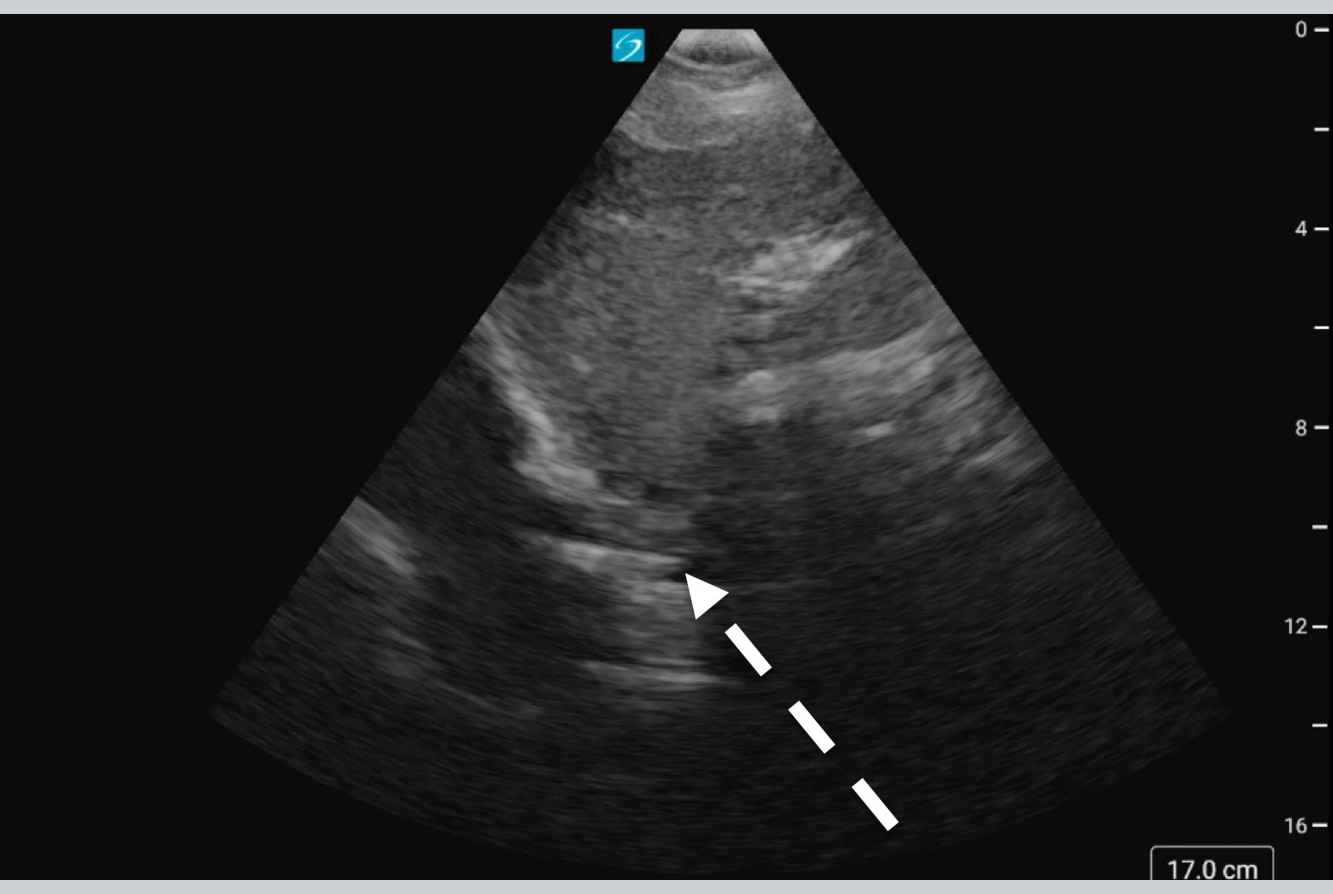
Subcostal point of care ultrasound (POCUS) window with the echogenic tip of transvenous pacing wire (dotted arrow) noted in the inferior vena cava (IVC) at the level of the cavoatrial junction (where the hepatic vein also meets with the IVC). The tip of the wire is directed inferiorly away from the right atrium. See Supplemental Video 4.

Next, we will review these locations and how they can be visualized using POCUS. The sonographer should first attempt to visualize the heart in another view, such as switching from subxiphoid to apical 4-chamber view or vice versa. If the pacing wire is unable to be visualized in a secondary view, the sonographer should return to the primary view and the clinician should slowly advance the wire to a depth of 25 cm. If the wire is not visualized at a depth of 25 cm, the sonographer should obtain a long-axis view of the IVC on ultrasound. This is accomplished by obtaining a sagittal view of the subxiphoid region and visualizing the IVC as it terminates into the right atrium, with the proximal hepatic vein as a landmark (Figure 6). If the pacemaker wire is seen in the proximal IVC, the wire should be retracted until it is no longer visualized on the IVC view. Then the sonographer may return to the prior cardiac view as the clinician re-advances the pacing wire. If needed, the wire may need to be completely retracted and given appropriate curvature into the right atrium.

If no wire is visualized in either the heart or IVC at 25 cm, we suggest continuing to slowly advance the wire to 30 cm while viewing the heart using POCUS. If the wire is not visualized in either the heart or IVC at 30 cm, we recommend inspecting the ipsilateral subclavian vessels with ultrasound. Misplacement into the subclavian vessels should be relatively rare, provided that the CVC is appropriately placed, and a real-time agitated saline flush is visualized in the right-side of the heart. However, as demonstrated by our first case above, misplacement can still occur. Visualization of these vessels can be accomplished by obtaining an infraclavicular view with the linear probe in a sagittal position on the distal clavicle. This view will allow visualization of both the subclavian vein and artery. If the distal clavicle is part of the sterile field, control of the ultrasound can be shifted back to the clinician; if the distal clavicle is not part of the sterile field, the sonographer will need to remove the linear probe from the sterile field (or alternatively, may use a second probe or ultrasound machine). If no vessels are visualized in the right subclavian region, the left subclavian vessels may be visualized in a similar manner. If the wire is not visualized in the heart, IVC, or subclavian vessels, the sonographer should next visualize the contralateral internal jugular vein via a transverse view of the lateral neck with the linear probe. If the wire is still not visualized, then the wire should be retracted and examined for a break or other mechanical faults.

## If the pacemaker wire is visualized but will not advance into the apex of the right ventricle

Placement of the wire in the right atrium is the most common location during TVP misplacement [5]. If the pacing wire is visualized in the heart but curls up in the right atrium or the right ventricle without advancing to the apex, the pacing wire can be retracted and rotated. If the wire is still not able to be advanced into the right ventricle, it can be completely retracted and given a leftward curvature and re-advanced. A curled TVP wire in the right atrium or ventricle may generate a pacing pattern on telemetry but may give an atrial pacing pattern that does not feature the characteristic LBBB pattern, may be inconsistent, or may require higher electrical output. Visualizing a curled wire in the right atrium or ventricle and readjusting wire placement with POCUS can prevent this.

## If sudden hemodynamic instability occurs

If the patient suddenly becomes unstable during the transvenous pacing procedure, POCUS can be used to identify several emergent conditions that may be associated with inadvertent vascular wire puncture. The sonographer should start by looking for a pericardial effusion that may be resulting in tamponade. This can be accomplished most accurately by obtaining a subxiphoid view and looking for an anechoic stripe surrounding the heart. Most frequently, this can be seen between the liver and the right ventricle. If no pericardial effusion is seen, the sonographer should attempt to rule out a pneumothorax by identifying lung sliding with the linear probe at the 2nd or 3rd midclavicular line starting with the right and then the left lung. If normal lung sliding is seen (i.e., no pneumothorax), then the sonographer should look for hemothorax by looking at the base of the lung, again starting with the right and then the left lung.

## Incorporating POCUS into a Standard TVP Workflow

The workflow described here has become commonplace in our department. Creating and refining this workflow came about because several physicians in our department had joint interest and training in both critical care and POCUS, as well as a number of cases for which TVP placement did not occur as smoothly as expected. POCUS is a standard tool used during our resuscitations and was a natural addition to the TVP procedure given the ability of POCUS to visualize the wire in cardiac views and the pre-existing use of POCUS to place a CVC. Our department also represents an academic, tertiary care referral center with a large catchment area. It is the primary clinical site for a three-year emergency medicine residency and sponsors fellowships in emergency ultrasound, resuscitation, and critical care, and we note that TVPs are relatively common procedures in our emergency department.

We understand that not everyone has a similar practice environment and that having three trained personnel in a single procedure may not be feasible for smaller community departments. We also acknowledge that the addition of POCUS may introduce additional challenges in high-stress clinical situations. These include when there is disagreement between POCUS and electrocardiogram readings (such as when there is successful electrical capture without clear POCUS confirmation of wire location), potential sub-optimal POCUS views due to a sonographer accounting for the sterile field and the additional cognitive load that POCUS may place on a clinician. But in our experience, the addition of POCUS to troubleshoot a wayward TVP wire can often lead to subsequent successful completion. The visualization of a pacemaker wire in the right ventricle serves as an excellent confirmatory test for proper TVP placement and can assuage clinical fears when a TVP placement does not go according to plan. Integrating POCUS into TVP placement can be tricky at first, but in our opinion, certainly gets easier with each instance of use.

## Conclusion

**Figure 7.**
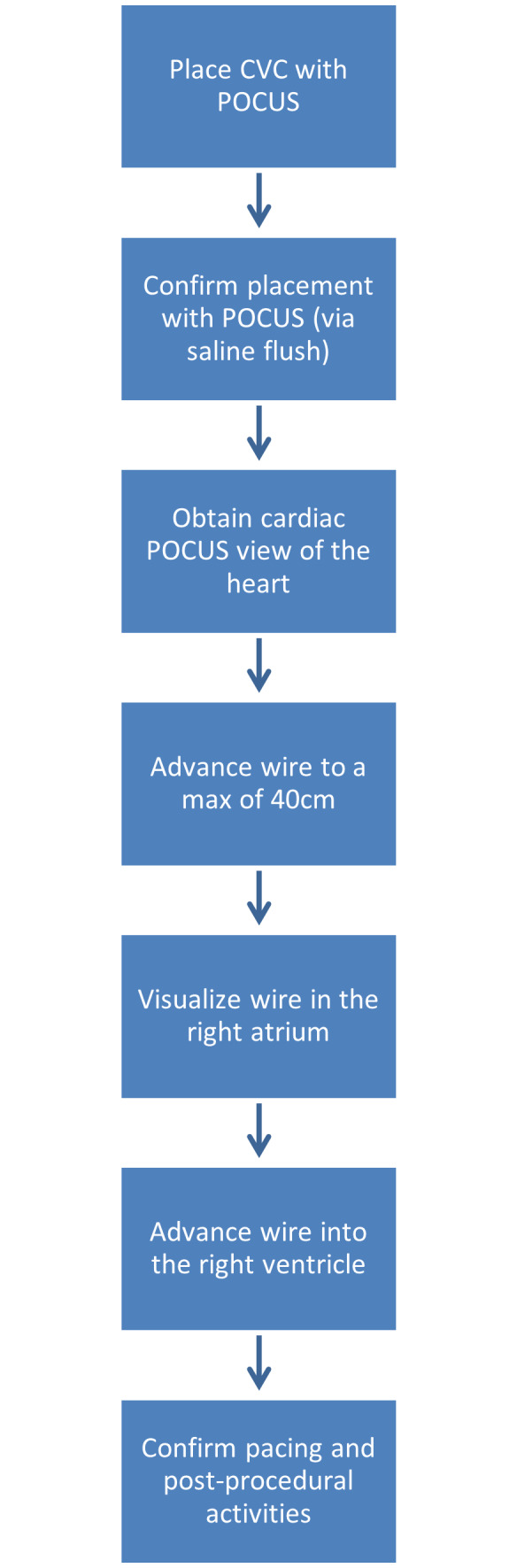
A flowchart demonstrating our current workflow incorporating point of care ultrasound (POCUS) into a normal, uncomplicated transvenous pacemaker (TVP) placement. CVC = central venous catheter.

**Figure 8.**
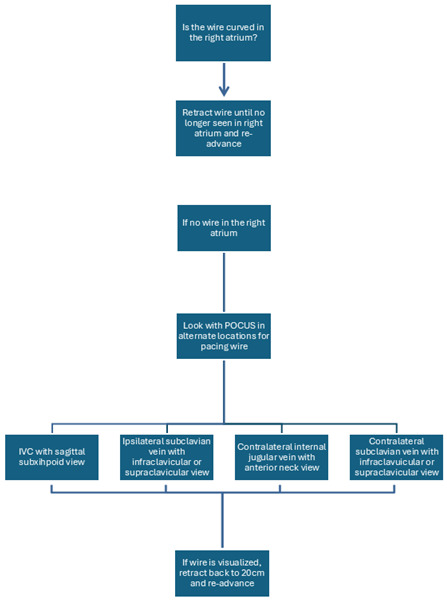
A flowchart demonstrating how point of care ultrasound (POCUS) can be used to troubleshoot transvenous pacemaker (TVP) placement should an issue arise during normal placement.

**Figure 9.**
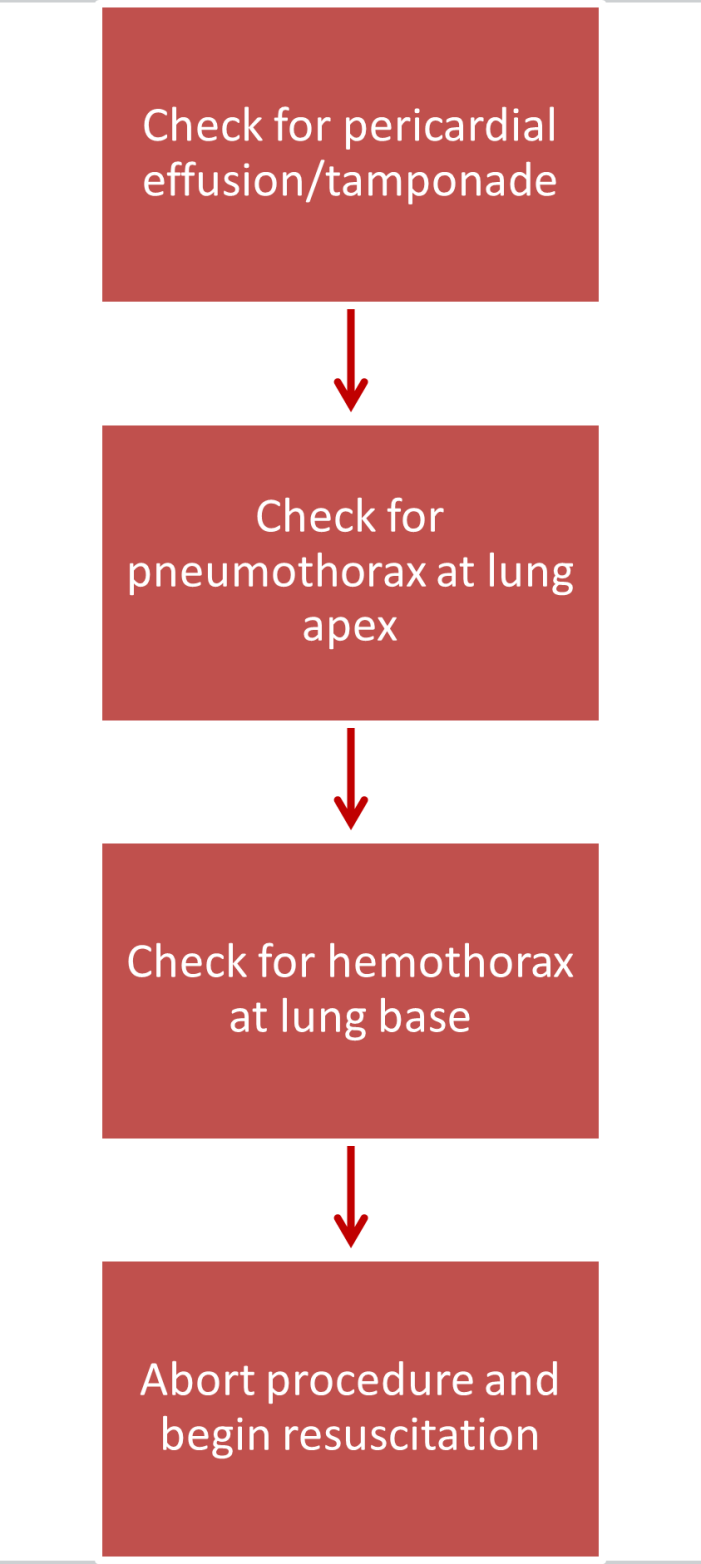
A flowchart demonstrating how to use point of care ultrasound (POCUS) should the patient become unstable during or immediately after transvenous pacemaker (TVP) placement.

POCUS is a useful tool in the placement of temporary TVPs, but it can also be a vital tool in troubleshooting complications. Our department's workflow in TVP placement implements ultrasound in both the placement of the CVC and the placement and advancement of the TVP wire. We share our normal workflow (Figure 7), a workflow for troubleshooting TVPs (Figure 8), and a workflow for if the patient becomes unstable (Figure 9). The workflows are also shared in table format (Table 1). Our workflow can be tailored and restructured to fit the needs of any department, but we suggest that POCUS have a more involved role in TVP procedures and be incorporated more frequently when these procedures acutely arise.

**Table 1.** This table incorporates Figures 7, 8 and 9 into an algorithmic manner.

The clinician should first place the central venous catheter (CVC) under standard point of care ultrasound (POCUS) guidance. The sonographer should then obtain a cardiac POCUS view that can be maintained through the procedure. The transvenous pacemaker (TVP) wire should be prepared and ad-vanced to a depth of 20 cm, after which the balloon should be inflated. As the TVP wire is slowly advanced to a depth of 35-40 cm, the TVP wire should be visualized in the right atrium. If the wire is not visualized in the right atrium: A retraction attempt can be made of the TVP wire back to 20 cm and then a slow re-advancement can be made. If the wire still is not visualized in the right atrium, alternate locations for the wire can be assessed for on POCUS: the inferior vena cava (IVC) and the subclavian veins. If the wire is located in one of these alternate areas, the wire can be slowly retracted under POCUS guidance and re-advanced. Once the wire is visualized in the right atrium, it should be slowly ad-vanced into the right ventricle under POCUS guidance. If the wire is curved in the right atrium and does not advance through the tricuspid valve, the wire can be retracted until no longer seen in the right atrium and re-advanced. Once the TVP wire is noted in the apex of the right ventricle and electri-cal capture is established, post-procedural activities may be conducted.








